# A latent class analysis of attitudes concerning the acceptability of intimate partner violence in rural Senegal

**DOI:** 10.1186/s12963-020-00233-0

**Published:** 2020-10-15

**Authors:** John Sandberg, Rosalind Fennell, Yacine Boujija, Laetitia Douillot, Valerie Delaunay, Simona Bignami, Wubin Xie, Cheikh Sokhna, Steven Rytina

**Affiliations:** 1 Milken Institute School of Public Health, Department of Global Health, The George Washington University, 950 New Hampshire Ave., NW, Washington, D.C., 20052 USA; 2 The George Washington University, Washington, D.C., USA; 3 Universite de Montreal, Montreal, Canada; 4 Laboratoire Population Environment Developpement, Institut de recherche pour le développement, Aix-Marseille Université, Marseille, France; 5 UMR VITROME, Institut de recherche pour le développement, Aix-Marseille Université, Dakar-Marseille, France; 6 Harvard University, Cambridge, USA

**Keywords:** Intimate partner violence, Measurement models, Latent class analysis, Attitudes, Norms

## Abstract

**Background:**

Research concerning the causes and consequences of intimate partner violence (IPV), particularly in less developed areas of the world, has become prominent in the last two decades. Although a number of potential causal factors have been investigated the current consensus is that attitudes toward IPV on the individual level, likely representing perceptions of normative behavior, and the normative acceptability of IPV on the aggregate level likely play key roles. Measurement of both is generally approached through either binary indicators of acceptability of any type of IPV or additive composite indexes of multiple indicators. Both strategies imply untested assumptions which potentially have important implications for both research into the causes and consequences of IPV as well as interventions aimed to reduce its prevalence.

**Methods:**

Using survey data from rural Senegal collected in 2014, this analysis estimates latent class measurement models of attitudes concerning the acceptability of IPV. We investigate the dimensional structure of IPV ideation and test the parallel indicator assumption implicit in common measurement strategies, as well as structural and measurement invariance between men and women.

**Results:**

We find that a two-class model of the acceptability of IPV in which the conditional probability of class membership is allowed to vary between the sexes is preferred for both men and women. Though the assumption of structural invariance between men and women is supported, measurement invariance and the assumption of parallel indicators (or equivalence of indicators used) are not.

**Conclusions:**

Measurement strategies conventionally used to operationalize the acceptability of IPV, key to modeling perceptions of norms around IPV, are a poor fit to the data used here. Research concerning the measurement characteristics of IPV acceptability is a precondition for adequate investigation of its causes and consequences, as well as for intervention efforts aimed at reducing or eliminating IPV.

## Background

In recent decades, there has been a growing call for the international community to address violence against women, and its elimination has been incorporated into the United Nations’ Sustainable Development Goals [1]. A robust research literature has developed concerning the causes and consequences of violence against women, particularly intimate partner violence (IPV), the physical, sexual, or emotional abuse of a woman by her spouse or partner [2]. It has been estimated that close to 1/3 of women around the world have experienced IPV, with a much higher prevalence in many less developed societies, particularly in sub-Saharan Africa [3, 4]. From a public health perspective, IPV has been linked to a variety of reproductive, physical, and mental health outcomes adversely affecting the well-being of women, their children, and their communities [2, 5–7].

Though the research and intervention literature concerning the causes of intimate partner violence have explored a number of potential structural, individual, and social factors potentially causally associated with IPV [8], a recent focus of this work has been on the influence of social norms concerning gender roles and the acceptability of spousal abuse [5, 6, 8–14]. Despite this current emphasis, relatively little work has addressed the measurement of such normative influence. Current operationalizations of norms associated with IPV are for the most part accomplished through aggregation of simple binary indicators or composite (generally additive) indexes of the experience of violence or beliefs and attitudes supporting it. Evidence that such measures are associated with the perpetration or experience of IPV is inconsistent, however [15–19]. This latter category, including beliefs or attitudes concerning the circumstances in which it is seen as acceptable for a male to physically abuse his partner, are likely not simply personally held, but perceptions of the prevailing normative context around IPV. As such, at the aggregate level such attitudes are potentially critical to understanding the normative contexts in which violence is (or is not) perpetrated [17, 20, 21]. To decrease measurement error associated with such aggregate measures necessitates careful attention to their measurement characteristics.

While possibly useful in some contexts, binary indicators and composite additive indexes are prone to error and discard information on variability that may be potentially vital to explaining both the causes and consequences of IPV. There is substantial variation in the specific circumstances under which individuals believe intimate partner violence is acceptable [8, 21]. A simple binary variable indicating the acceptability of violence in any scenario does not indicate acceptability in all, or indeed, any more than that single one. An additive index, while utilizing more information, ignores variation in the degree to which different circumstances are seen as acceptable and potentially important information concerning the distribution of different combinations of these. Both types of measures give equal weight in indicating the underlying latent construct to all indicators, implicitly employing what is known as the assumption of parallel indicators. Both types of measures erase exactly the nuances in normative cultural models of IPV that may make explanation more precise and which may be exploited by interventions aimed at reducing its prevalence.

## Methods

The present analysis, using data from a rural population in Senegal, estimates a series of latent class measurement models of the acceptability of physical violence against wives. Latent class analysis (LCA) is a standard method for identifying latent class or group membership using categorical indicators [22, 23]. Since norms are powerful in motivating and justifying behavior because of their instantiation in particular members of a population of interacting individuals, classifying members of such a population based on their heterogeneous attitudes toward spousal abuse is an appropriate approach to this problem. The models estimated here specify five indicators of acceptability under different, hypothetical scenarios, widely available in the Demographic and Health Surveys (DHS). These indicators are commonly used to measure attitudes on individual level and, on the aggregate level, the normative context of IPV.

If ideation concerning violence against wives is unidimensional, we would expect only two classes of individuals, one for those supportive of spousal violence, one for those who are not, or potentially a multiple-class result where the probability of class membership conditional on each item was ordered. In either case, all indicators will be reflective of the underlying dimension of support for spousal violence, albeit not necessarily equally so. A multidimensional result, in contrast, would be revealed by different classes of individuals supportive of different combinations of situations in which spousal violence is deemed acceptable.

Following the literature having identified gendered differences in beliefs supportive of IPV in which women are often seen to be more accepting of it than men [8, 18, 19, 21], we also test structural and measurement invariance between men and women in a multi-group analysis. Finally, we test the parallel indicator assumption that the weight attributed to each scenario in indicating the underlying latent construct of approval of IPV is equal, implicit in binary or composite index measures.

### Data

The data used for this analysis come from the first panel of the Niakhar Social Networks and Health Project (NSNHP), a large-scale longitudinal social network survey collected in 2014 in collaboration with the Niakhar Demographic and Health Surveillance System (NDHSS) [24]. The NDHSS, maintained by the French national development research agency *l’Institute de Recherché pour le Développment* (IRD) and located approximately 150 km south east of Dakar in Senegal’s Siin region, has prospectively monitored demographic and health events for the entire populations of 30 contiguous villages since 1982. The majority of the study area’s current population of 44,000 identifies as ethnically Sereer (94%) and Muslim (78%). However, a significant proportion of the study zone population is Christian (approximately 20.8%). The region’s economy is largely rooted in small livestock and agricultural production of millet and peanuts [25].

The NSNHP main survey panel includes a large, representative sample of the population age 16 and older of the NDHSS catchment area (*n* = 882), as well as a census of the entire population of one town in the area, Yandé (*N* = 1310). The response rate for the survey was above 95%. For the purposes of this analysis, population weights are used to ensure representativeness of the entire surveillance zone, which, when applied, yield an analytic weighted sample size of 945 (399 men and 546 women) including 879 respondents from the population outside of Yandé and the weighted census equal to 66 respondents from Yandé. The survey contained a large module concerning a number of health and demographic topics including, importantly for this research, acceptability of intimate partner violence under different scenarios.

Respondents were asked five questions derived from the 2014 Senegal DHS concerning the acceptability of a husband beating his wife under different scenarios. These included (1) if she goes out without telling him, (2) if she neglects the children, (3) if she disagrees with him, (4) if she refuses to have sex with him, and (5) if she burns the food. Respondents were asked to respond “yes” or “no,” indicating if they found it acceptable for a husband to beat his wife in the presented scenario. For each of these situations, a dichotomous variable was created with response categories as 0 “no” or 1 “yes.” For the purposes of this analysis, all unknown and nonresponses were coded as missing. This missing data was relatively rare, ranging from a maximum of 2.5% of responses to the indicator concerning refusal of sex to 0.2% for that concerning burning food.

### Latent class analysis

Using responses to the five indicators of the acceptability of IPV under the scenarios described above, we first estimate models for 2, 3, and 4 classes for the population as a whole using robust maximum likelihood estimation and population weights to ensure representativeness. To test structural invariance between men and women (that men and women have the same preferred number of latent classes), we estimate these same solutions for men and women separately. We next test for measurement invariance, or the assumption that item thresholds and latent class probabilities are equal between men and women in the preferred structural solution. Finally, we test the parallel indicator assumption in each set of models estimated by constraining the conditional probability of class membership associated with all indicators to be equal and comparing the fit of the constrained and unconstrained specifications. Estimation was performed through full-information maximum likelihood (FIML), which accumulates casewise likelihood across the entire sample assuming data are missing at random (MAR). This yields unbiased estimates that are more efficient than casewise or listwise deletion strategies [26]. All models were estimated with 1200 random starts to avoid local maxima and ensure replication of the best loglikelihood, in MPLUS v7.3 [27].

## Results

**Fig. 1:**
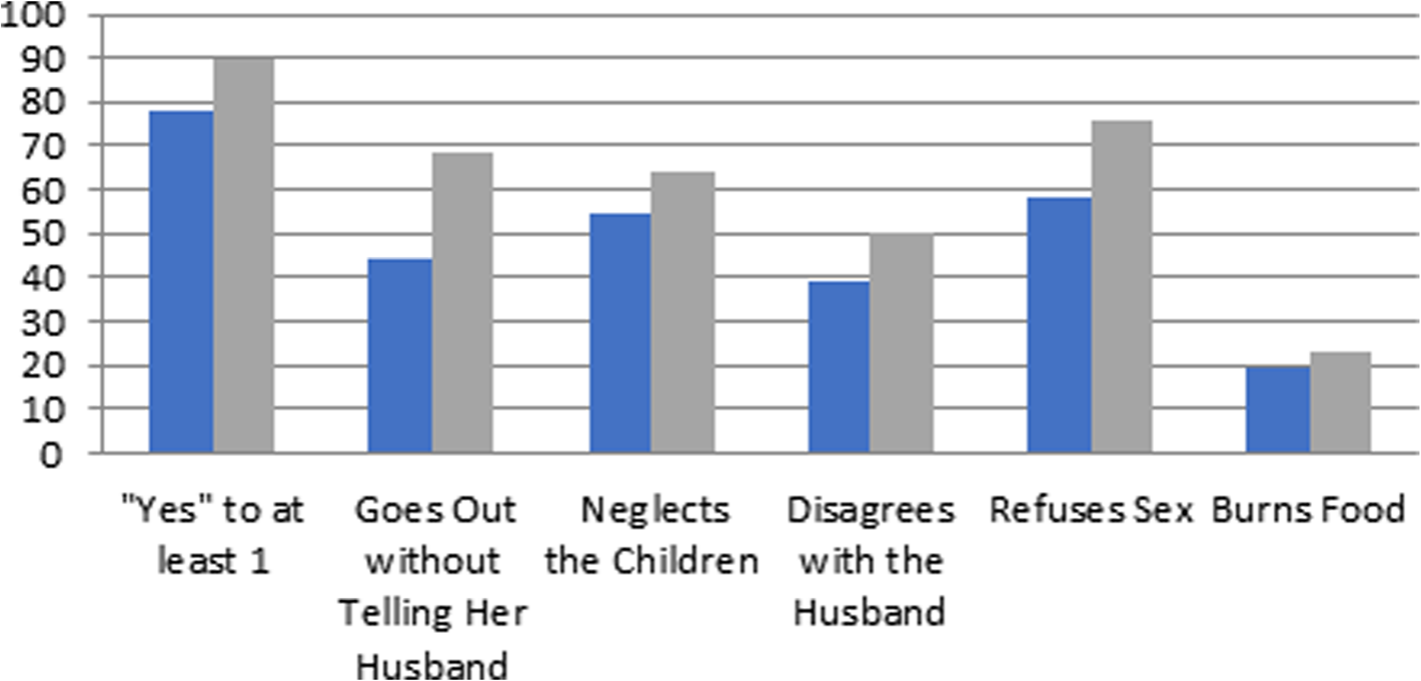
Percentages of men and women finding beating wife acceptable, by scenario, NDHSS zone (*n* = 945). Blue (first bar): men who answered “yes.” Grey (second bar): women who answered “yes”

Figure 1 presents the estimated percentages of men and women in the sample finding spousal violence acceptable for each of the five indicators in the present analysis and for at least one. Overall, the level of acceptability of spousal violence in this population is high. As expected, women indicate the acceptability of spousal violence at higher rates than men across all scenarios. The scenario that both men and women find most acceptable is refusal of sex, with almost 60% of men and 75% of women answering yes to this question. The least acceptable scenario is that for when the wife burns food, with only about 20% of men and women finding this an acceptable reason for a husband to beat his wife. The difference between acceptability in these two scenarios underscores the wider variance within the population across all scenarios presented. All rationales for spousal abuse do not appear to have equal support. This is to be expected but has potentially important implications for empirical analyses that measure approval of violence where different measures are assumed to have equal weight in indicating underlying support, as in the construction of indexes, or as a binary construct where individuals approve of spousal violence for at least one of these reasons. This latter operationalization is shown in the first set of bars in Fig. 1. Almost 80% of men and 90% of women find at least one of these rationales for spousal violence acceptable.

Table 1 presents fit statistics for the latent class models of the acceptability of spousal abuse estimated. Traditional unconditional measures of model fit here are the AIC and BIC, with lower values indicating better fit. The Vuong-Lo-Mendell Rubin (VLMR-LR) and bootstrapped LR (BLRT) tests are likelihood ratio tests appropriate for testing the relative fit of latent class models of k-1 vs. k classes [28]. The latter, however, was not estimable using population weights in MPLUS, and we present only the VLMR-LR test. Though by the unconditional criteria, the three-class model would be preferred, by the VLMR test, the two-class model is clearly preferred here. Under this model, 64% of the population is classified as more supportive of spousal violence, 36% as (relatively) unsupportive. Neither class is estimated to find beating wives for burning food acceptable, however, and there is a not insubstantial likelihood of supporting the other rationales for violence among the unsupportive class. This suggests a more nuanced picture of ideation concerning spousal violence than is often acknowledged.

**Table 1:** Latent class model fit, acceptability of spousal violence among adults age 16+, NDHSS study area (*n* = 945)

Model	Number of latent classes (free parameters)	***G***^**2**^	AIC	BIC	***l***	VLMR-LR (df)
**Total population**						
1	2 (11)	54.669	12,325.860	12,388.458	− 6151.93	1420.31 (6) (*p* < 0.0000)
2	3 (17)	25.795	12,285.578	12,382.320	− 6125.78	52.28 (6) (*p* = 0.3471)
3	4 (23)	14.543	12,278.315	12,409.202	− 6116.15	19.26 (6) (*p* = 0.4115)
1a	2 (3)	99.092	13,999.531	14,016.603	− 6996.76	971.27 (2) (*p* < 0.0000)
**Females only**						
4	2 (11)	50.038	6666.031	6722.104	− 3322.01	653.61 (6) (*p* < 0.0000)
5	3 (17)	26.339	6638.444	6725.103	− 3302.22	39.587 (6) (*p* = 0.5015)
6	4 (23)	14.075	6630.850	6748.098	− 3292.42	− 3302.222 (*p* = 0.6303)
4a	2 (3)	343.661	7789.406	7804.698	− 3891.70	403.297 (2) (*p* = 0.0000)
**Males only**						
7	2 (11)	26.714	5520.832	5574.584	− 2749.41	695.095 (6) (*p* < 0.0000)
8	3 (17)	17.125	5513.994	5597.065	− 2739.99	18.839 (6) (*p* = 0.5464)
9	4 (23)	11.812	5516.192	5628.582	− 2735.09	9.802 (6) (*p* = 0.5708)
7a	2 (3)	72.583	6111.212	6125.872	− 3052.60	491.782 (2) (*p* < 0.0000)
**Multi-group males/females**						
10	2 (23)	77.915	15,160.661	15,291.549	− 7557.33	–
11 (invariant)	2 (11)	316.338	16,475.675	16,538.273	− 8226.83	–
10a	2 (7)	153.141	16,892.909	16,932.745	− 8439.45	–

Source: NSNHP Panel 1, 2014: compiled by the author

Models 4, 5, and 6 and 7, 8, and 9 estimate the same models for women and men separately, testing the assumption of structural invariance. In both sets of models, we see the same result as in those for the population as a whole. A two-class model is preferred by the VLMR test.

**Fig. 2:**
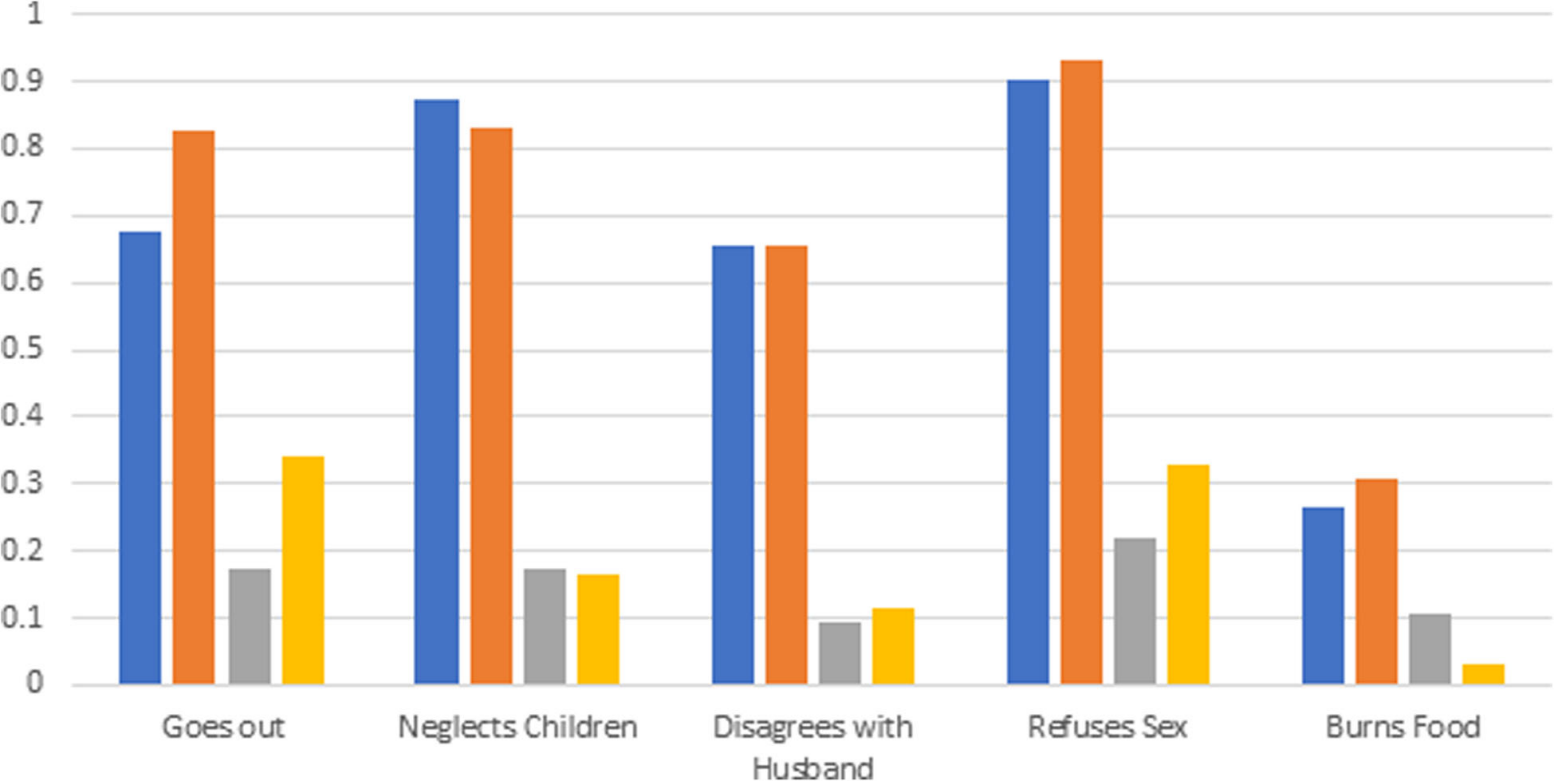
Estimated conditional probability of class membership by sex, model 10, NDHSS zone (*n* = 945). Blue (first bar): class 1 males. Orange (second bar): class 1 females. Grey (third bar): class 2 males. Yellow (third bar): class 2 females

Having established structural invariance between men and women, we proceed to test measurement invariance, the assumption that the conditional probabilities of class membership for the indicators are equal for men and women. Model 10 presents fit statistics for a multi-group model in which the probabilities of class membership conditional on observed responses are allowed to be different for men and women. Model 11 constrains them to be equal across the two groups. In this case, the information criteria are unequivocal. Model 10, where men and women have different probabilities of their respective class membership across the indicators is preferred by the model fit criteria shown here. In combination with the results above concerning structural invariance, this provides strong evidence that although both men and women may be classified into two groups, supportive and relatively unsupportive, they differ significantly in degree to which particular spousal abuse scenarios drive that support (or lack thereof). The estimated probabilities of class memberships under this model are presented in Fig. 2. Class 1, comprised of 53% of men and a stunning 71% of women, with a high degree of within-class homogeneity, is generally supportive of spousal violence, most prominently for refusal of sex and neglect of children. Women in this class are slightly more likely to find spousal violence for going out without permission and refusal of sex acceptable, men slightly more likely than women to find violence justified by neglect of children acceptable. The second class, with the remaining 46% of men and 29% of women, is generally unsupportive of spousal violence, but still somewhat so, especially in the cases of going out without permission and refusal of sex. Women classified as unsupportive are much more likely to say they find these justifications acceptable than men. In comparison, the three and four class models for both men and women show a pronounced lack of separation between the additional classes estimated. For women, both models split the supportive class seen in the preferred model into two separate classes, one only slightly less supportive than the other. The four-class solution adds to this a marginal class comprised of less than 2% of women who are unsupportive of abuse for going out without permission, but universally supportive of abuse for burning food. For men, we see a similar pattern. The three-class solution, while retaining an almost identical supportive class, splits the less supportive class into 2 very close unsupportive classes, one with slightly less support than the other. The four-class solution is almost identical, with the addition of a fourth class with less than 5% of the sample classified as completely unsupportive of abuse under any scenario.

Finally, the fit statistics in models 1a, 4a, 7a, and 10a refer to models in which the indicators in the preferred model from each panel are constrained such that each indicator has the same estimated conditional probability of class membership. Comparison of these models to their unconstrained analogs provides a test of the parallel indicator assumption. In each case, the constrained models fit demonstrably worse than the unconstrained models, providing strong evidence against this assumption.

In sum, the preferred measurement model in this analysis is model 10. This solution suggests that in this population, acceptability of spousal violence is unidimensional, in that all indicators are a reflection of the same binary construct, for men and women alike. Multidimensional solutions with separate classes more supportive of violence under certain circumstances (for example, poor performance of household activities or childcare or insufficient deference to male authority) did not fit the data as well. It also suggests, however, that men and women are not identical in the way these indicators reflect this underlying dimension.

## Discussion

Causal factors currently believed to be associated with intimate partner violence include, prominently, individual attitudes and social norms concerning its acceptability. Prior substantive research concerning both has largely employed simple operationalizations, including binary indicators of any acceptability and additive composite indexes of this complex latent construct with untested measurement properties and assumptions. In this paper, we have tested a series of measurement models of attitudes concerning the acceptability of intimate partner violence under five scenarios, indicators that are widely available through the Demographic and Health Surveys (DHS) and other sources and commonly used in the literature. In the rural Senegalese population from which our sample was drawn, we find support for a two-class model, where one class of respondents was generally supportive of intimate partner violence, the other less so. In this model, the estimated degree of support for IPV in each class varied across the indicators, however, and was found to be different for men and women.

What this analysis has demonstrated is that in this population, common measurement strategies fit the data poorly relative to our preferred model. Both simple binary indicators of acceptability under any scenario and additive indexes assume each indicator carries equal weight in the underlying latent construct of IPV, which our results definitively indicate is untenable in this context. Both measurement strategies ignore variation in acceptability under different circumstances, which we have shown to be substantial here.

Our results should be qualified in at least two important ways. First, we have modeled only a limited set of indicators here. Analyses incorporating different indicators, such as those related to the experience of IPV, or, when investigating norms, perceptions of the acceptability of IPV among others, may, and quite likely would, yield different preferred measurement models. Second, generalizations drawn from this analysis are limited to this particular rural population. We have no reason to believe a priori that this model would be appropriate for other populations, even within sub-Saharan Africa. We believe the first limitation is justifiable in that the indicators we have chosen here are commonly used to operationalize both attitudes and, on the aggregate level, norms, concerning IPV in the current research literature and that investigation of their measurement properties as part of a unique latent construct is warranted for this reason. We also believe that the lack of a priori generalizability to other populations is a desirable feature of this analytic approach. Given our knowledge of variation in attitudes concerning the acceptability of intimate partner violence both within and between populations, we should not expect one structural and measurement model to fit them all, an assumption made with the use of conventional measures.

## Conclusion

Investigation of variation in the measurement of IPV acceptability, from population to population, between subgroups within populations (defined by, for example, ethnicity and religion among other factors), is necessary to both the understanding of cultural constructions of the acceptability of IPV and accurate representations of the causal structure behind both attitudes and norms related to it as well as their consequences. Though as noted above, the research literature has suggested a number of possible associations between IPV and critical public health outcomes, this evidence generally tends to be tentative. More definitive results will depend on, among other things, more accurate measurement strategies than have previously been employed.

Those engaged in intervention work would also undoubtedly benefit from more accurate measurement, potentially allowing them to develop and tailor more efficient programming and achieve improved results. Researchers and practitioners in public health would do well to address the latent structure and measurement characteristics of these types of indicators (as well as others they employ) in each specific case if we are to, as a discipline, efficiently achieve the goal of reducing the prevalence of such attitudes and associated norms and through them intimate partner violence. We have shown in other research in this population using the measurement model estimated here, for example, how potentially normative influence through social network interaction shapes the acceptability of spousal violence, suggesting specific intervention strategies exploiting this influence [23].

Our research suggests that simple binary measures indicating the acceptability of any type of spousal violence, and likely, any type or frequency of violence should be avoided, as should simple additive indexes of these. In addition, though we found no evidence for it here, in other populations important differences may exist with regard to the dimensionality of ideation supportive of or the perpetration of violence. Some violence, for example, may be justified by violation of gendered role expectations as in the case of domestic chores or childcare, some by factors associated with patriarchal norms concerning the control and seclusion of women. Some cultural schemas and institutions supporting each of these hypothetical dimensions may be more amenable to change through specific types of intervention, some more amenable to others. Exploiting knowledge of such differences would likely lead to more effective and efficient interventions. To date latent methods (such as latent class or factor analysis) capable of identifying such dimensionality, assessing the relative weight of particular constructs in indicating them, and testing for inter-group (such as gender or ethnic) differences have not been widely used for this. There is no reason they cannot be, however, given their availability in all popular statistical software applications and the wealth of data measuring violence and the ideation supporting it across a variety of sources such as the DHS.

## Data Availability

The dataset analyzed in the current study is available from the corresponding author on application and approval for appropriate scientific use by the IRD, Senegal, and the NSNHP. Details on application may be obtained from the corresponding author.

## Abbreviations

IPV: Intimate partner violence
DHS: Demographic and Health Survey
NSNHP: Niakhar Social Networks and Health Project
NDHSS: Niakhar Demographic and Health Surveillance System
IRD: l’Institute de Recherché pour le Développment

## References

[1] García-Moreno C, Amin A. The sustainable development goals, violence and women’s and children’s health. Bulletin of the World Health Organization. 2016;94:396–7. 10.2471/BLT.16.172205 PMC4850543 27147771

[2] García-Moreno C, Zimmerman C, Morris-Gehring A, Heise L, Amin A, Abrahams N (2015). Addressing violence against women: a call to action. Lancet..

[3] Devries KM, Mak JYT, García-Moreno C, Petzold M, Child JC, Falder G, et al. The Global Prevalence of Intimate Partner Violence Against Women. Science. 2013;340:1527–8. 10.1126/science.1240937 23788730

[4] World Health Organization. Global and Regional Estimates of Violence Against Women: Prevalence and Health Effects of Intimate Partner Violence and Non-partner Sexual Violence. World Health Organization; 2013.

[5] Abramsky T, Watts CH, Garcia-Moreno C, Devries K, Kiss L, Ellsberg M (2011). What factors are associated with recent intimate partner violence? Findings from the WHO multi-country study on women’s health and domestic violence. BMC Public Health.

[6] Arango DJ, Morton M, Gennari F, Kiplesund S, Ellsberg M. Interventions to prevent or reduce violence against women and girls: a systematic review of reviews. The World Bank; 2014. http://documents.worldbank.org/curated/en/700731468149970518/Interventions-to-prevent-or-reduce-violence-against-women-and-girls-a-systematic-review-of-reviews. Accessed 28 Sep 2016.

[7] Hindin MJ, Kishor S, Ansara DL (2008). Intimate partner violence among couples in 10 DHS countries: predictors and health outcomes.

[8] Heise L. What works to prevent partner violence? An evidence overview. 2011. http://strive.lshtm.ac.uk/resources/what-works-prevent-partner-violence-evidence-overview. Accessed 28 Sep 2016.

[9] Bott S, Morrison A, Ellsberg M. Preventing and Responding to Gender-based Violence inmiddle and low-income countries: a global review and analysis. Washington, DC: World Bank Publications; 2005.

[10] Hossain M, Zimmerman C, Kiss L, Abramsky T, Kone D, Bakayoko-Topolska M (2014). Working with men to prevent intimate partner violence in a conflict-affected setting: a pilot cluster randomized controlled trial in rural Côte d’Ivoire. BMC Public Health.

[11] Jewkes R, Flood M, Lang J (2015). From work with men and boys to changes of social norms and reduction of inequities in gender relations: a conceptual shift in prevention of violence against women and girls. Lancet..

[12] McCleary-Sills J (2013). Jordanian social norms and the risk of intimate partner violence and limited reproductive agency. J Int Womens Studies.

[13] Michau L, Horn J, Bank A, Dutt M, Zimmerman C (2015). Prevention of violence against women and girls: lessons from practice. Lancet..

[14] Pierotti RS (2013). Increasing rejection of intimate partner violence evidence of global cultural diffusion. Am Sociol Rev.

[15] Hayes BE, Boyd KA (2017). Influence of individual- and national-level factors on attitudes toward intimate partner violence. Sociol Perspect.

[16] Okenwa-Emegwa L, Lawoko S, Jansson B. Attitudes Toward Physical Intimate Partner Violence Against Women in Nigeria. SAGE Open. 2016;6:2158244016667993..

[17] Tsai AC, Kakuhikire B, Perkins JM, Vořechovská D, McDonough AQ, Ogburn EL (2017). Measuring personal beliefs and perceived norms about intimate partner violence: population-based survey experiment in rural Uganda. PLoS Med.

[18] Uthman OA, Lawoko S, Moradi T (2009). Factors associated with attitudes towards intimate partner violence against women: a comparative analysis of 17 sub-Saharan countries. BMC Int Health Human Rights.

[19] Speizer IS (2010). Intimate partner violence attitudes and experience among women and men in Uganda. J Interpersonal Violence.

[20] Heise L, Kotsadam A (2015). Cross-national and multilevel correlates of partner violence: an analysis of data from population-based surveys. Lancet Global Health.

[21] Kishor S, Subaiya L (2008). Understanding women’s empowerment: a comparative analysis of demographic and health surveys (DHS) data.

[22] Hagenaars JA (1990). Categorical longitudinal data log-linear panel, trend.

[23] Goodman LA. Latent class analysis: the empirical study of latent types, latent variables, and latent structures. Appl Latent Class Analysis. 2002. 10.1017/CBO9780511499531.002.

[24] Delaunay V, Douillot L, Rytina S, Boujija Y, Bignami S, Gning SB, et al. The Niakhar social networks and health project. MeX. 2019. https://doi.org/10.1016/j.mex.2019.05.037. 10.1016/j.mex.2019.05.037 PMC6580193 31431893

[25] Delaunay V. La situation démographique dans l’Observatoire de Niakhar : 1963-2014. Dakar: IRD; 2017. http://www.documentation.ird.fr/hor/fdi:010071521.

[26] Enders CK, Bandalos DL (2001). The relative performance of full information maximum likelihood estimation for missing data in structural equation models. Struct Equ Model Multidiscip J.

[27] Linda K. Muthén, Bengt O. Muthen. Mplus User’s Guide. 7th edition. Los Angeles: Muthén & Muthén; 1998.

[28] Nylund KL, Asparouhov T, Muthén BO (2007). Deciding on the number of classes in latent class analysis and growth mixture modeling: a Monte Carlo simulation study. Struct Equ Model Multidiscip J.

